# Cancer Care in Africa: An Overview of Resources

**DOI:** 10.1200/JGO.2015.000406

**Published:** 2015-09-23

**Authors:** Daniela Cristina Stefan

**Affiliations:** South African Medical Research Council, Cape Town, South Africa.

## Abstract

**Purpose:**

According to predictions from the International Agency for Research on Cancer, over the next 5 years, the annual number of new cases of cancer in Africa will grow to more than one million. Together with the immense loss in human life, there is a considerable economic setback attached to this number. However, most African nations are far from adequately scaling up their capacity to control cancer.

**Methods:**

This study reviews the published data on the existing cancer control resources in Africa. It is, to our knowledge, the first combined effort looking at all resources available on the continent regarding cancer care.

**Results:**

The total number of 102 cancer treatment centers, including general oncology centers, gynecologic oncology or other single-organ malignancy units, and pediatric oncology and palliative care establishments, is not sufficient to cover the increasing needs of the African population affected by cancer. In addition, the continental average total health expenditure per capita amounts to only US$82.

**Conclusion:**

This review could serve as a starting point for devising realistic solutions meant to improve the prevention and management of malignant disease on the African continent.

## INTRODUCTION

Survival of people with cancer in Africa is far worse than that attained in high-income countries. For example, the 5-year survival rate of women with breast cancer in Europe is 82%, whereas it is 46% in Uganda, a little less than 39% in Algeria, and 12% in Gambia.^1^

Numerous teams of researchers have dissected the issue in the last decade, with the aim of identifying the causes and proposing solutions for these poor outcomes. Although the research published so far has highlighted many of the shortcomings of cancer care and enunciated valid strategic principles, there has been no setting of targets and time lines on a realistic path to achieve the desired progress.

The Brazzaville Declaration on Noncommunicable Diseases Prevention and Control in the WHO African Region, adopted in 2011,^2^ affirmed the awareness of African governments about the increasing health danger from noncommunicable diseases (NCDs), including cancers. Among others, the signatories undertook to develop strategies for prevention and control, to strengthen their health systems to enable them to reduce the burden of NCDs, to source the finances required for fighting these diseases, and to enable their national health information systems to generate data on NCDs and their risk factors. However, the progress in this direction is still slow. In 2010, a survey initiated by WHO in the African region found that of 46 respondent countries, only 17 had operational policies, strategies, or action plans for cancer. The few existing formal national cancer control plans (NCCP) were not funded yet.^3^ In 2013, according to yet unpublished data (J.M. D'Angou, 2013 survey), of 32 African countries, only 11 had a formal NCCP, and in 17 other countries, such plans were still being prepared. Of the 11 existing NCCPs, only six were driven by a steering committee, which would be a requirement for adequate functioning.

Realistic plans to control cancer must take into account the extent of the health problem, the prevailing types of cancers, and their distribution by region, age group, and sex. Such data can only be provided by well-run national population-based cancer registries, yet there are too few such institutions on the continent. According to the African Cancer Registry Network, in the sub-Saharan Africa region, only 22 member registries contribute to the network's database, and not all of them are national registries.^4^ The recorded data quality level is not yet of the required standard in many of the African registries; as a result, only five were accepted to contribute to the International Agency for Research on Cancer's (IARC) periodic publication *Cancer Incidence in Five Continents* (volume IX).^5^

In the absence of precise figures, the burden of cancer can only be estimated. GLOBOCAN 2012, the latest estimation of cancer incidence, prevalence, and mortality in the world done by the IARC, calculated that in 2012 just less than 850,000 new cancers appeared in Africa, while in the same year, almost 600,000 deaths were attributed to malignant disease. The predictions for 2020 are approximately 1,056,000 new cases (an increase of approximately 24%) and more than 735,000 deaths. In 2012 in Africa, the mortality/incidence ratio, an expression of the efficacy of cancer care systems, was 72%, considerably higher than the ratio in high-income populations; in Europe, for example, it was 44%.^6^ The aim of this article is to provide a comprehensive evaluation of the resources for cancer treatment and palliation in Africa based on the published information available to date.

## METHODS

Whenever possible, an attempt was made to include data for the whole continent, not only for the WHO African Region.^7^ However, most of the literature focuses only on sub-Saharan Africa, and those findings cannot be extrapolated to North African countries.

The data on incidence and mortality, as well as the predictions for 2020, were extracted from GLOBOCAN 2012: Estimated Cancer Incidence, Mortality and Prevalence Worldwide in 2012, which is available on the Internet from the IARC. The geographic location of radiotherapy installations in Africa was found in the 2013 analysis of the International Atomic Energy Agency (IAEA). The distribution of pathology services over sub-Saharan Africa was found in a recently published analysis. The financial information was extracted from the database of the World Bank.^8^

A literature search was performed on the Medline database using PubMed with the following search terms: cancer AND any of the following: chemotherapy, radiotherapy, control, expenditure, prevention, palliation, pathology, centers, medicines, surgeons AND Africa. The references lists of selected reports were scrutinized for relevant titles. Similar searches were run on Google and Google Scholar. Preference was given to the most recent review publications and analyses. In addition, the WHO, International Union Against Cancer, and Economist Intelligence Unit Web sites were explored for any items related to cancer control in Africa.

**Figure 1:**
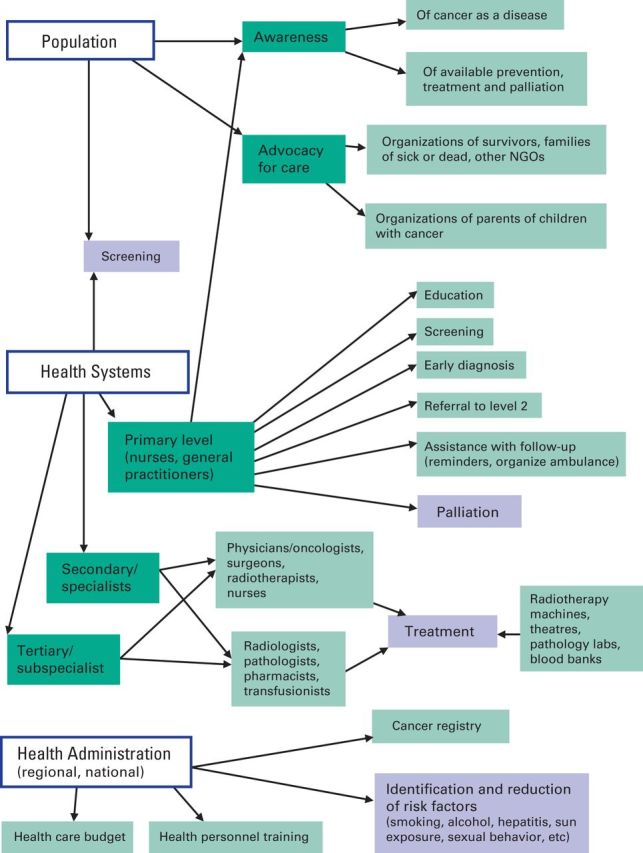
Diagram of a cancer control system. NGO, nongovernmental organization.

To assist with focusing the analysis of existing resources, estimating services needed in the present and future, and framing the discussions in this article and in the following articles, a diagram of the cancer care system components and their interconnections was drawn (Fig 1).

## RESULTS

### Comparative Incidence Rates of Cancers in Africa

In the absence of reliable data available from national cancer registries, most statistics on cancer in Africa are estimates only. According to GLOBOCAN 2012, the highest estimated incidence rates were for breast and uterine cervix cancers in females and prostate cancer in males. Other frequently encountered malignancies on the continent were lung, liver, colorectal, and esophageal cancer, non-Hodgkin lymphoma, Kaposi's sarcoma, and skin cancer. The overall crude annual incidence of cancer in the African population was estimated at79 cases per 100,000 population.

### Oncology Centers and Palliation

Although a comprehensive list of all cancer treatment facilities in Africa remains to be compiled, a valuable step in the right direction was made by the African Organization for Research and Training in Cancer. In an effort to foster collaboration and coordination between the various institutions working for cancer control on the continent, the African Organization for Research and Training in Cancer launched the African Cancer Network Project in 2012. As part of the project, a list was published, composed of cancer treatment, research, teaching, advocating, fund-raising, and administrative entities in Africa. The list, a work in progress, contains 102 cancer treatment institutions, including general oncology centers, gynecologic oncology or other single-organ malignancy units, and pediatric oncology and palliative care establishments. Of these institutions, 38 are located in South Africa.^9^ Although the list is not yet complete, it suggests that a massive undersupply of cancer care services exists on the continent.

Even if comprehensive cancer control remains the ideal worthy of striving for, palliative care can be achieved at a lower cost and ensures that much of the suffering associated with advanced cancers is controlled. WHO has recently published the results of a worldwide survey of the status of palliative care for all diseases, including cancer palliation activities. There was no evidence of any palliation activity for adults in 22 African countries (although WHO admitted that some activities may take place). In five countries, capacity building was under way; 16 countries provided palliation in certain regions only; two countries offered generalized palliation services; and in five countries, the activity was at a preliminary stage of integration with the mainstream health services.^10^

With regard to palliative care for children, the WHO survey could not provide any data for most of Africa. Capacity building is occurring in five countries, whereas in another country, there is localized delivery of palliation. Palliative activities are integrated in the main health care system in only one country. The WHO survey estimated the number of adults and children with cancer in need of palliative care in Africa at more than 360,000.^10^

Within the palliative care armamentarium, analgesia has a central place. Pain was found to be highly prevalent in patients with cancer; in a study of 112 patients with cancer, as many as 87.5% reported suffering from pain.^11^ WHO considers morphine to be an essential drug in combating pain, including pain from advanced malignant disease.^10^ A patient requiring palliative pain treatment would use, on average, 67.5 mg of morphine daily for approximately 3 months. Generally, the cost of this treatment is modest; for example, in Uganda, it was calculated that the total cost for morphine for one patient over 3 months would amount to $23.^12^

Even so, the annual opioid (expressed as morphine equivalent) consumption in sub-Saharan Africa was reported to amount to 720 kg from 2007 to 2009, which would be enough to treat approximately 120,000 patients (ie, approximately one third of those requiring palliative care). When the South African consumption of 71% of the total is factored in, it is clear that most of the patients with cancer who need pain relief are not receiving it.

### Surgical Oncology

An official evaluation of the number of oncology surgeons or general surgeons in Africa could not be found. In 2010, a comprehensive review of the literature originating in sub-Saharan Africa (excluding South Africa) on the state of surgery in the region^13^ revealed that the number of surgeons was approximately less than two surgeons per 100,000 inhabitants. For comparison, the same number in England was greater than 35 surgeons. The consequence was that, in numerous district hospitals, surgery and anesthesia were (and still are) carried out by nonphysician personnel, who underwent some form of training to perform those tasks. In referral hospitals, most of the oncologic surgery was performed by general surgeons.^14^ The main reasons for the low number of surgeons in the region, according to the study, were the massive emigration of medical school graduates and the absence of surgical specialization programs in many African countries.

### Radiotherapy

How many radiotherapy units exist at present in Africa? Are these adequately covering the need for cancer treatment? How many more units will be required in 2020 or later? Much of the work required to answer these questions has already been done by the IAEA, as will be presented here.

Radiotherapy plays a major role in the curative armamentarium of a cancer unit. When looking at the principal therapeutic means used to cure patients with cancer, surgery was found to constitute the main method in 49% of patients, radiation in 40% of patients, and chemotherapy in the remaining 11% of patients.^15^ In the high-income country of Australia, a survey found that a little more than 52% of patients with cancer needed radiotherapy as a component of their therapeutic plan.^16^ However, in low-income countries, it is estimated that, because of insufficient surgery services and the large percentage of advanced-stage cancers that require palliative treatment, up to 60% to 70% of new patients require radiotherapy.^16^

Against this background, in 2010, the Directory of Radiotherapy Centres maintained by the IAEA registered only 277 external-beam radiotherapy machines for the African continent. Sixty percent of these were located in two countries—South Africa and Egypt. At the same time, 29 African countries (of a total of 52 surveyed) did not provide radiotherapy to patients with cancer. IAEA has calculated that, considering the annual incidence of cancer in Africa of 713,206 patients (estimation made in GLOBOCAN 2008) and also the treatment capacity of 450 new cancers per year for one single teletherapy machine, more than 700 additional such machines would be required on the continent.^17^

Notwithstanding the validity of these calculations, one needs to observe that the underlying assumptions are that all patients with a newly presenting cancer would access the health care system and that all of them would be diagnosed correctly. An additional assumption, made also for the purpose of this study, would be that the estimations presented in GLOBOCAN are close to the real figures. Unfortunately, at present and for some time to come, a large fraction of patients with a newly presenting cancer in Africa do not access the health services.

### Availability of Chemotherapy

There are no published exact data on the availability of chemotherapeutic medicines for the whole of Africa. An evaluation of the situation for sub-Saharan Africa was attempted in 2012^18^ and highlighted a number of difficulties. Although it is likely that all 22 chemotherapeutic drugs on the WHO essential list are imported in the region, most of them as generics, not all of the medicines are available all the time. By extrapolating the situation of other essential medicines on the WHO list, whose availability was shown to cover only half of the demand, it is presumed that there is a considerable shortage of systemic anticancer agents. Moreover, the authors found that, generally, the prices of medicines were between 2.7 and 6.1 times higher in Africa than the international reference prices. Finally, they estimated that there are too few trained doctors and nurses able to administer the chemotherapy.

An analysis of the availability of cancer therapy drugs at a cancer center in Tanzania illustrates the situation^19^; only approximately 50% of the specific medicines were available during the period surveyed, which led to more than 70% of patients not receiving adequate therapy. Buying the drugs privately cost an amount equivalent to between 1 and 7 months of income. Because most of the patients were not insured, only a few were able to pay.

### Pathology Services

Both anatomic and clinical pathology services are indispensable for cancer control. From screening for malignant disease, through diagnosis, staging, guiding the surgical act, and evaluating the complications of management, to monitoring the effect of treatment, every step along the way is supported by pathology investigations. Moreover, cancer registry data can only be credible if supported by pathologic confirmation of the diagnoses. This last contribution is essential for a realistic planning of cancer control strategies.

Although there are no official data on the state of pathology services in Africa, the available information points to a major deficit, both in volume and quality. An informal survey of pathology capacity in sub-Saharan Africa, completed in 2012,^20^ indicated that, by rapport with the population numbers, the number of pathologists in the region amounted to approximately 10% of the number of similar specialists in high-income countries. The insufficient numbers of pathologists and technicians, the poor condition of the equipment, the suboptimal infrastructure, and the difficulty in obtaining the laboratory supplies were also acknowledged at the last African Pathologists Summit held in 2013 in Dakar.^21^

### Prevention

The literature on cancer prevention in Africa was comprehensively reviewed in a recently published study.^22^ The review iterates that the highest incident cancers in Africa are, largely, preventable. For example, cervical cancer can be prevented by population vaccination against the human papillomavirus and various population screening methods; liver cancer can be prevented by vaccination against hepatitis B virus; the risk for Burkitt lymphoma can be reduced by eradicating malaria and HIV infections (the latter is also the main contributor to the high incidence of Kaposi's sarcoma); and skin cancer can be prevented by minimizing exposure to sunlight. However, the review found that the preventive measures in the region are inadequate. The literature indicates that there is insufficient population awareness of the existence of cancer as a disease, of cancer's risk factors and manifestations, and of the modalities for prevention and cure of cancer. Moreover, various cultural factors hinder the application of preventative measures that have been devised for other sociocultural environments. The health budgets and health care infrastructure and personnel are insufficient to support mass prevention activities. Vaccination campaigns against hepatitis B virus and human papillomavirus take place on the continent, but they are mainly financed from external sources such as the Global Alliance for Vaccines and Immunizations or United Nations organizations. Governments do not have the will to engage fully in cancer control, and most countries lack national cancer registries, which would be a source of reliable data on the extent and nature of the problem.

### Financing

The Economist Intelligence Unit, in an analysis of the global burden of cancer published in 2009, found a large disparity between the African share of 15% of the world population, its share of 6.4% of the world's annual new cases of cancer, and the cancer costs on the continent, amounting only to 0.3% of the global costs.^23^ Because the main component of cancer costs is represented by the medical expenditure (medicines, medical procedures, costs of hospitalization, and outpatient visits), it follows that the amount spent on cancer treatment is disproportionately low in Africa.

This finding is not unexpected. An analysis of the health financing in the WHO African Region found that although in 2001 the African governments adopted the Abuja Declaration, whereby they committed to increasing their expenditure on health to 15% of their national budgets, only four countries had fulfilled that promise by 2009. In the same year, in 21 African countries, more than 50% of the total health expenditure was covered by private sources, and in 26 countries, more than 70% of the private expenditure on health was disbursed out of pocket.

Moreover, many African countries rely heavily on foreign donations, either directly to the health budget or for specific health care projects. The contribution of external donor money to health care financing increased from 2001 to 2009, with 19 countries relying on foreign aid to cover more than 20% of their total health expenses. This financing source is potentially insecure, given the global economic crisis; often the funds are earmarked for certain purposes and cannot be used in other ways.^24^

Under these circumstances, the continental average total health expenditure per capita amounted only to US$82.^25^ The figure is certainly above the US$34 indicated by WHO as the minimum expenditure per capita required for providing basic primary health care. However, this amount clearly cannot fund comprehensive secondary and tertiary health services. For comparison, in the same year, the health expenditure per capita in the United Kingdom was US$3,512.^26^

## DISCUSSION

Over the last few years, an increasing number of researchers have attempted to investigate the causes and remedies for the inadequate cancer control in Africa. The reason for this increasing preoccupation lies in the changing spectrum of diseases on the continent; successes in controlling infectious diseases, coupled with an improvement in the conditions of living, have led to an increase in lifestyle-related NCDs, including cancer. These changes were observed decades ago in higher income countries and are observed today in Africa. The trend will continue, resulting in a considerable increase in the incidence of cancers in a relatively short period. An additional increase in some types of cancer, such as non-Hodgkin lymphomas and Kaposi's sarcoma, is a result of the HIV/AIDS epidemic.

The resources available for cancer control are less than adequate in Africa, and the reasons for this are complex and huge. They start with a lack of awareness of the disease in large sections of the population and, hence, a weak advocacy for adequate political action and are compounded by the absence of reliable national statistical data on cancer. This lack of data allows governments to procrastinate on creating cancer control plans or, if such plans are already in place, to delay providing the necessary funds. The lack of funds is a direct consequence of the modest national income per capita. Many countries in Africa rely on foreign aid for attaining their health care goals and are far from fulfilling their Abuja promise of allocating 15% of their national budgets to health care. Against this background, the infrastructure and personnel dedicated to cancer care and prevention are disproportionately small. Moreover, palliative care is often left to philanthropic organizations, and even their work is hindered by the massive lack of opiates for pain control. The import and use of these medicines are frequently over-restricted, far beyond the reasonable safety requirements.

This review focused on the shortcomings of the cancer control systems, with the aim of charting a way toward achieving the capacity to cope with the inevitable increase in malignant disease in Africa. Remarkable achievements have occurred already, and some of those will be discussed in a future overview. 
